# Supplementary material for: A Likelihood Approach for Real-Time Calibration of Stochastic Compartmental Epidemic Models
Source: PLoS Comput Biol. 2017 Jan 17;13(1):e1005257. doi: 10.1371/journal.pcbi.1005257 (PMC5240920; doi:10.1371/journal.pcbi.1005257)
Supplement: S1 File — (TAR.GZ) [file pcbi.1005257.s014.tar.gz › HSPH_Online-SI-Revision/EnKF07/n100-extreme/n100-extreme_EnKF2_table8.pdf]

1	newly 3 weeks spec	655.	2427.41	{73., 6945.}	346.328	0.588507	newly 3 weeks cumul	2650.	11 624.	{906., 53 786.}	385.583	0.521796	newly 5 weeks spec	298.	1019.58	{8., 3010.}	335.436	0.802528	newly 5 weeks cumul	3391.	14 186.8	{1023., 59 732.}	367.978	0.515972	newly 46 weeks cumul	4195.	17 116.9	{1130., 73 475.}	360.687	0.52167
2	newly 3 weeks spec	761.	1395.73	{0., 2238.}	199.686	1.46576	newly 3 weeks cumul	3077.	17 879.	{2353., 57 548.}	493.936	0.553813	newly 5 weeks spec	441.	150.07	{0., 706.}	92.5828	2.22462	newly 5 weeks cumul	4068.	18 444.2	{2353., 62 064.}	368.006	0.470704	newly 42 weeks cumul	5247.	18 598.2	{2353., 62 451.}	271.907	0.394806
3	newly 3 weeks spec	277.	1807.69	{519., 2111.}	554.278	0.530963	newly 3 weeks cumul	1321.	6666.53	{1931., 8075.}	407.173	0.477542	newly 5 weeks spec	122.	1098.53	{215., 1236.}	801.402	0.608418	newly 5 weeks cumul	1607.	9177.26	{2736., 10 973.}	473.165	0.510624	newly 47 weeks cumul	1853.	12 737.8	{3847., 14 049.}	588.827	0.567723
4	newly 3 weeks spec	75.	2261.35	{124., 4552.}	2915.29	1.12626	newly 3 weeks cumul	495.	12 529.3	{3707., 16 304.}	2431.17	1.16971	newly 5 weeks spec	26.	979.18	{4., 1666.}	3686.85	1.22062	newly 5 weeks cumul	564.	14 969.9	{3804., 23 337.}	2554.24	1.18675	newly 51 weeks cumul	589.	17 433.	{3805., 35 914.}	2859.77	1.23088
5	newly 3 weeks spec	383.	2056.56	{0., 2118.}	490.627	1.13166	newly 3 weeks cumul	1884.	12 712.6	{2084., 21 791.}	576.308	0.563471	newly 5 weeks spec	164.	1485.67	{0., 1427.}	864.348	1.42745	newly 5 weeks cumul	2324.	15 977.6	{2624., 46 843.}	588.992	0.57391	newly 48 weeks cumul	2679.	20 588.3	{3420., 86 599.}	670.204	0.611017
6	newly 3 weeks spec	620.	2500.06	{0., 5377.}	374.245	1.49974	newly 3 weeks cumul	2591.	21 376.7	{4395., 35 412.}	729.694	0.732279	newly 5 weeks spec	303.	1011.66	{0., 1848.}	335.432	1.50121	newly 5 weeks cumul	3314.	23 872.9	{5062., 40 391.}	625.061	0.675899	newly 46 weeks cumul	4005.	26 544.6	{5594., 59 963.}	567.579	0.636668
7	newly 3 weeks spec	425.	1174.09	{53., 2626.}	228.064	0.560372	newly 3 weeks cumul	2093.	14 527.9	{3475., 19 917.}	601.524	0.579386	newly 5 weeks spec	238.	161.2	{1., 209.}	97.8739	0.961072	newly 5 weeks cumul	2658.	15 099.5	{3481., 28 593.}	476.11	0.503781	newly 44 weeks cumul	3121.	15 263.4	{3482., 33 739.}	397.614	0.447516
8	newly 3 weeks spec	611.	2737.44	{0., 5227.}	434.147	1.62476	newly 3 weeks cumul	2634.	19 645.3	{1765., 34 139.}	660.463	0.748866	newly 5 weeks spec	315.	1094.26	{0., 1831.}	352.324	1.66903	newly 5 weeks cumul	3371.	22 444.8	{1776., 37 432.}	582.339	0.708446	newly 46 weeks cumul	4133.	24 854.1	{1778., 38 483.}	519.21	0.674434
9	newly 3 weeks spec	509.	798.73	{54., 1921.}	132.969	0.476713	newly 3 weeks cumul	2442.	17 877.5	{1048., 65 150.}	651.794	0.668836	newly 5 weeks spec	256.	51.97	{1., 145.}	86.8242	1.35049	newly 5 weeks cumul	3053.	18 128.5	{1062., 67 649.}	516.749	0.611691	newly 46 weeks cumul	3616.	18 149.8	{1062., 67 887.}	427.502	0.567909
10	newly 3 weeks spec	32.	733.47	{0., 920.}	2267.53	1.56246	newly 3 weeks cumul	165.	11 376.	{1603., 18 388.}	6794.56	1.581	newly 5 weeks spec	7.	358.87	{0., 294.}	5114.43	1.39243	newly 5 weeks cumul	184.	12 215.2	{1614., 23 305.}	6538.71	1.56094	newly 50 weeks cumul	192.	13 813.8	{1614., 30 183.}	7094.7	1.56997
11	newly 3 weeks spec	582.	2444.62	{7., 11 778.}	422.137	0.935405	newly 3 weeks cumul	2565.	13 755.1	{478., 49 238.}	483.39	0.475407	newly 5 weeks spec	318.	895.37	{0., 922.}	300.544	1.39306	newly 5 weeks cumul	3307.	16 058.7	{539., 59 049.}	440.047	0.48639	newly 44 weeks cumul	4044.	18 145.9	{553., 68 900.}	413.459	0.532955
12	newly 3 weeks spec	488.	1367.77	{85., 2854.}	235.17	0.463367	newly 3 weeks cumul	2113.	17 552.3	{1595., 83 293.}	740.289	0.617941	newly 5 weeks spec	223.	92.33	{2., 236.}	68.6413	0.881063	newly 5 weeks cumul	2630.	17 963.5	{1861., 86 209.}	593.403	0.549456	newly 45 weeks cumul	3083.	18 191.1	{2238., 86 312.}	499.609	0.493213
13	newly 3 weeks spec	1177.	2015.21	{0., 5718.}	181.382	1.28902	newly 3 weeks cumul	4644.	11 438.3	{4512., 30 514.}	155.67	0.292956	newly 5 weeks spec	679.	1181.7	{0., 4031.}	213.564	1.66559	newly 5 weeks cumul	6187.	14 120.8	{4570., 33 518.}	147.15	0.271009	newly 43 weeks cumul	8302.	18 780.8	{4570., 58 796.}	169.176	0.334119
14	newly 3 weeks spec	9.	3364.67	{0., 8251.}	37 323.	2.0049	newly 3 weeks cumul	64.	17 195.7	{490., 23 422.}	26 770.2	1.92055	newly 5 weeks spec	5.	2008.58	{0., 9874.}	40 112.	2.12691	newly 5 weeks cumul	74.	21 799.9	{762., 45 084.}	29 360.8	1.99784	newly 48 weeks cumul	86.	28 971.6	{1828., 90 161.}	33 589.4	2.14075
15	newly 3 weeks spec	73.	1679.06	{74., 2418.}	2215.26	1.10983	newly 3 weeks cumul	382.	6689.1	{1717., 9752.}	1651.07	1.01017	newly 5 weeks spec	19.	1292.8	{1., 4706.}	6732.21	1.39101	newly 5 weeks cumul	449.	9478.26	{2269., 18 869.}	2010.97	1.04866	newly 48 weeks cumul	476.	15 114.3	{2434., 60 510.}	3075.27	1.13793
16	newly 3 weeks spec	111.	2135.27	{112., 5503.}	1831.11	0.963477	newly 3 weeks cumul	653.	9011.31	{2862., 14 337.}	1281.87	0.91667	newly 5 weeks spec	31.	1471.83	{28., 6910.}	4665.19	1.28229	newly 5 weeks cumul	748.	12 176.5	{4257., 27 425.}	1529.64	0.974144	newly 48 weeks cumul	812.	19 982.3	{4983., 86 570.}	2362.58	1.06958
17	newly 3 weeks spec	848.	1560.14	{9., 5098.}	180.509	0.802665	newly 3 weeks cumul	3379.	11 144.2	{938., 23 660.}	250.155	0.363686	newly 5 weeks spec	459.	680.21	{0., 2324.}	177.571	1.33811	newly 5 weeks cumul	4480.	12 797.	{986., 29 794.}	212.393	0.334005	newly 43 weeks cumul	5685.	16 496.	{1295., 50 105.}	228.029	0.369693
18	newly 3 weeks spec	371.	1082.68	{6., 2307.}	284.652	0.799386	newly 3 weeks cumul	1789.	10 426.9	{2184., 34 529.}	496.567	0.510048	newly 5 weeks spec	168.	101.45	{0., 213.}	107.351	1.41637	newly 5 weeks cumul	2207.	10 849.7	{2184., 35 048.}	406.119	0.447612	newly 44 weeks cumul	2601.	10 905.9	{2184., 35 081.}	335.068	0.394991
19	newly 3 weeks spec	522.	2109.34	{30., 7381.}	367.456	0.739979	newly 3 weeks cumul	2300.	15 285.2	{1392., 50 122.}	579.781	0.649624	newly 5 weeks spec	225.	945.22	{0., 2714.}	408.258	1.27703	newly 5 weeks cumul	2858.	17 632.3	{1548., 50 413.}	532.62	0.614014	newly 46 weeks cumul	3344.	19 704.3	{1592., 68 496.}	505.479	0.586393
20	newly 3 weeks spec	31.	1813.78	{6., 5194.}	5777.48	1.07762	newly 3 weeks cumul	168.	5202.47	{81., 16 536.}	3015.1	1.02833	newly 5 weeks spec	4.	2019.69	{0., 10 833.}	50 425.3	1.55237	newly 5 weeks cumul	185.	9462.49	{82., 52 443.}	5032.9	1.0995	newly 51 weeks cumul	187.	13 805.9	{89., 77 573.}	7299.78	1.17458
21	newly 3 weeks spec	203.	1842.03	{0., 3852.}	849.404	1.18009	newly 3 weeks cumul	1027.	8026.2	{3000., 11 947.}	683.363	0.714956	newly 5 weeks spec	76.	1701.82	{0., 7941.}	2184.45	1.48052	newly 5 weeks cumul	1233.	11 515.3	{3733., 27 835.}	835.701	0.768963	newly 48 weeks cumul	1341.	19 300.	{4035., 82 639.}	1340.39	0.882557
22	newly 3 weeks spec	138.	658.62	{0., 1610.}	499.797	2.02381	newly 3 weeks cumul	788.	11 008.8	{1392., 28 174.}	1299.72	0.831925	newly 5 weeks spec	76.	306.23	{0., 793.}	433.013	2.12607	newly 5 weeks cumul	953.	11 755.3	{1392., 28 174.}	1137.45	0.79059	newly 49 weeks cumul	1046.	12 506.8	{1392., 28 174.}	1100.34	0.780937
23	newly 3 weeks spec	646.	1962.18	{0., 5186.}	306.04	1.1716	newly 3 weeks cumul	2717.	22 118.5	{6294., 76 149.}	714.541	0.664006	newly 5 weeks spec	343.	394.45	{0., 784.}	167.169	2.02817	newly 5 weeks cumul	3563.	23 349.4	{6640., 85 855.}	555.99	0.564875	newly 42 weeks cumul	4442.	23 763.8	{6640., 93 374.}	435.822	0.475934
24	newly 3 weeks spec	888.	494.36	{13., 1543.}	72.6667	0.965156	newly 3 weeks cumul	3507.	6784.5	{1047., 10 657.}	124.88	0.377615	newly 5 weeks spec	441.	92.84	{0., 461.}	82.4354	1.84543	newly 5 weeks cumul	4589.	7084.31	{1051., 10 920.}	89.1891	0.329739	newly 42 weeks cumul	5895.	7176.83	{1051., 10 950.}	67.2246	0.314
25	newly 3 weeks spec	135.	367.32	{0., 462.}	275.615	1.14463	newly 3 weeks cumul	801.	6557.57	{2247., 10 015.}	725.172	0.76883	newly 5 weeks spec	46.	77.22	{0., 151.}	212.304	1.66699	newly 5 weeks cumul	936.	6796.58	{2602., 10 015.}	633.269	0.720675	newly 45 weeks cumul	1018.	6880.93	{2723., 10 015.}	583.5	0.693397
26	newly 3 weeks spec	1048.	1844.16	{0., 8125.}	201.868	1.7445	newly 3 weeks cumul	4173.	10 619.1	{2988., 26 893.}	176.854	0.353903	newly 5 weeks spec	644.	1121.93	{0., 3447.}	225.443	2.30759	newly 5 weeks cumul	5651.	13 188.2	{3161., 42 219.}	174.957	0.378716	newly 43 weeks cumul	7533.	16 067.6	{3169., 50 346.}	173.617	0.423345
27	newly 3 weeks spec	806.	3844.27	{137., 13 280.}	430.713	0.664971	newly 3 weeks cumul	3332.	22 929.5	{1244., 57 528.}	618.642	0.675373	newly 5 weeks spec	451.	1514.58	{4., 6483.}	313.849	0.848777	newly 5 weeks cumul	4401.	26 866.5	{1727., 73 321.}	542.993	0.643464	newly 44 weeks cumul	5811.	30 244.5	{2176., 88 956.}	455.904	0.610591
28	newly 3 weeks spec	568.	2941.58	{98., 9935.}	475.271	0.592201	newly 3 weeks cumul	2482.	10 039.3	{562., 22 734.}	345.676	0.521275	newly 5 weeks spec	266.	2014.	{10., 10 423.}	735.85	0.825273	newly 5 weeks cumul	3132.	14 465.7	{684., 46 050.}	404.477	0.545281	newly 48 weeks cumul	3853.	22 099.7	{783., 89 091.}	519.239	0.588557
29	newly 3 weeks spec	769.	670.35	{9., 1594.}	70.7867	0.620868	newly 3 weeks cumul	3241.	11 993.	{4697., 28 980.}	273.586	0.402864	newly 5 weeks spec	412.	89.09	{0., 231.}	78.5558	1.51901	newly 5 weeks cumul	4213.	12 305.	{4902., 29 808.}	196.43	0.314169	newly 42 weeks cumul	5339.	12 378.9	{4939., 29 869.}	140.606	0.240346
30	newly 3 weeks spec	278.	621.82	{76., 724.}	180.353	0.400309	newly 3 weeks cumul	1314.	4380.95	{1392., 3635.}	239.126	0.294595	newly 5 weeks spec	87.	185.8	{6., 337.}	198.414	0.645153	newly 5 weeks cumul	1559.	4898.83	{1447., 4392.}	221.779	0.275909	newly 51 weeks cumul	1722.	5198.61	{1450., 4819.}	214.5	0.279671
31	newly 3 weeks spec	58.	2854.43	{222., 8633.}	4821.43	1.19085	newly 3 weeks cumul	360.	9598.19	{1477., 17 172.}	2566.16	1.06937	newly 5 weeks spec	22.	1876.55	{57., 8997.}	8432.14	1.242	newly 5 weeks cumul	413.	13 834.6	{1640., 37 701.}	3249.77	1.11956	newly 52 weeks cumul	442.	18 642.8	{1695., 80 580.}	4117.82	1.16307
32	newly 3 weeks spec	101.	1679.21	{184., 1812.}	1563.75	0.761535	newly 3 weeks cumul	549.	6697.4	{954., 7046.}	1120.52	0.716202	newly 5 weeks spec	30.	811.44	{53., 1122.}	2606.47	0.89592	newly 5 weeks cumul	633.	8684.4	{1268., 9539.}	1272.68	0.74508	newly 49 weeks cumul	682.	10 619.4	{1377., 21 632.}	1457.91	0.782416
33	newly 3 weeks spec	918.	2484.4	{173., 5344.}	199.728	0.401164	newly 3 weeks cumul	3832.	8796.25	{732., 14 097.}	161.766	0.400067	newly 5 weeks spec	479.	1802.48	{144., 6481.}	317.035	0.507873	newly 5 weeks cumul	4959.	12 721.	{937., 25 938.}	185.044	0.406249	newly 47 weeks cumul	6261.	21 708.3	{2067., 75 037.}	272.201	0.456675
34	newly 3 weeks spec	843.	213.23	{0., 854.}	84.7106	2.51827	newly 3 weeks cumul	3518.	4362.84	{1907., 6159.}	66.5117	0.229258	newly 5 weeks spec	471.	50.23	{0., 167.}	89.6709	2.58568	newly 5 weeks cumul	4638.	4515.07	{1955., 6159.}	57.6514	0.257307	newly 42 weeks cumul	6028.	4588.16	{1955., 6291.}	52.722	0.295837
35	newly 3 weeks spec	693.	2465.74	{121., 5107.}	277.775	0.569553	newly 3 weeks cumul	3121.	9317.06	{434., 13 048.}	223.488	0.560693	newly 5 weeks spec	338.	1616.03	{86., 3069.}	401.411	0.592365	newly 5 weeks cumul	3977.	12 920.7	{601., 25 286.}	248.192	0.569766	newly 47 weeks cumul	4760.	19 893.9	{1294., 52 878.}	337.881	0.610669
36	newly 3 weeks spec	635.	2021.11	{45., 3160.}	300.893	0.643991	newly 3 weeks cumul	2904.	10 668.5	{355., 23 205.}	305.76	0.470322	newly 5 weeks spec	364.	627.64	{1., 726.}	187.407	1.01822	newly 5 weeks cumul	3816.	12 433.1	{421., 25 447.}	274.59	0.468311	newly 45 weeks cumul	4844.	13 216.2	{540., 26 074.}	232.342	0.474713
37	newly 3 weeks spec	121.	1082.25	{18., 1280.}	832.868	0.753813	newly 3 weeks cumul	634.	13 901.2	{3800., 44 316.}	2094.38	1.08054	newly 5 weeks spec	49.	234.38	{0., 436.}	463.673	1.09054	newly 5 weeks cumul	759.	14 610.9	{4225., 44 462.}	1826.78	1.02765	newly 50 weeks cumul	824.	14 929.	{4226., 44 471.}	1713.49	1.00587
38	newly 3 weeks spec	12.	1295.24	{86., 1007.}	10 694.	1.61706	newly 3 weeks cumul	113.	6576.83	{599., 6487.}	5720.96	1.47274	newly 5 weeks spec	3.	436.64	{16., 582.}	14 461.3	1.72617	newly 5 weeks cumul	121.	7743.71	{821., 6666.}	6300.4	1.52036	newly 50 weeks cumul	126.	8609.86	{1410., 6906.}	6733.86	1.56718
39	newly 3 weeks spec	302.	1746.36	{168., 5506.}	495.152	0.553411	newly 3 weeks cumul	1471.	18 348.7	{6327., 38 479.}	1147.36	0.882131	newly 5 weeks spec	115.	297.2	{4., 544.}	224.696	0.626043	newly 5 weeks cumul	1796.	19 337.2	{6464., 41 160.}	976.683	0.815882	newly 44 weeks cumul	2002.	19 606.8	{6481., 49 735.}	879.442	0.773341
40	newly 3 weeks spec	529.	676.74	{1., 435.}	176.662	1.38405	newly 3 weeks cumul	2325.	7404.19	{2147., 23 465.}	226.396	0.274952	newly 5 weeks spec	275.	174.84	{0., 152.}	137.855	2.514	newly 5 weeks cumul	2963.	7920.76	{2233., 24 122.}	187.884	0.251159	newly 45 weeks cumul	3697.	8119.41	{2234., 24 122.}	158.812	0.268901
41	newly 3 weeks spec	253.	2186.36	{0., 3428.}	834.656	1.48944	newly 3 weeks cumul	1228.	14 979.5	{3167., 19 084.}	1123.79	0.827371	newly 5 weeks spec	114.	1158.2	{0., 1977.}	1002.11	1.5552	newly 5 weeks cumul	1511.	17 713.7	{3313., 23 182.}	1076.34	0.802633	newly 46 weeks cumul	1685.	21 647.7	{3362., 39 844.}	1188.69	0.822976
42	newly 3 weeks spec	23.	2178.18	{333., 3907.}	9374.87	1.71538	newly 3 weeks cumul	166.	12 979.8	{3786., 16 285.}	7725.22	1.76213	newly 5 weeks spec	8.	1051.	{12., 1409.}	13 043.8	1.50226	newly 5 weeks cumul	186.	15 507.5	{4618., 19 271.}	8243.09	1.77873	newly 50 weeks cumul	187.	18 091.3	{5134., 31 927.}	9579.32	1.8203
43	newly 3 weeks spec	681.	1073.56	{0., 4383.}	192.543	1.35132	newly 3 weeks cumul	3118.	7616.14	{682., 24 706.}	186.814	0.36235	newly 5 weeks spec	377.	442.83	{0., 1475.}	171.	1.92689	newly 5 weeks cumul	4043.	8738.21	{891., 29 778.}	171.129	0.370701	newly 43 weeks cumul	5018.	9963.46	{1129., 31 809.}	167.825	0.404723
44	newly 3 weeks spec	174.	493.26	{44., 534.}	210.195	0.380477	newly 3 weeks cumul	824.	7293.22	{822., 6979.}	796.017	0.728187	newly 5 weeks spec	61.	58.58	{1., 78.}	96.7213	0.686556	newly 5 weeks cumul	988.	7518.19	{838., 7088.}	673.195	0.677961	newly 49 weeks cumul	1084.	7552.57	{840., 7099.}	609.645	0.647774
45	newly 3 weeks spec	45.	1923.55	{124., 2144.}	4177.53	1.21172	newly 3 weeks cumul	244.	8205.2	{1395., 10 151.}	3263.8	1.22746	newly 5 weeks spec	9.	1372.45	{23., 4182.}	15 162.3	1.57484	newly 5 weeks cumul	276.	11 151.	{2080., 15 389.}	3940.92	1.29113	newly 48 weeks cumul	280.	19 034.1	{3839., 82 400.}	6698.	1.43804
46	newly 3 weeks spec	223.	1574.96	{2., 1430.}	641.65	0.898588	newly 3 weeks cumul	1040.	8103.89	{1828., 16 423.}	679.538	0.669618	newly 5 weeks spec	61.	1154.68	{0., 1195.}	1834.16	1.23995	newly 5 weeks cumul	1223.	10 614.6	{2770., 25 620.}	768.051	0.693227	newly 48 weeks cumul	1322.	15 521.4	{3207., 54 427.}	1074.36	0.750765
47	newly 3 weeks spec	132.	3242.88	{206., 5818.}	2360.61	1.13194	newly 3 weeks cumul	614.	26 417.	{2290., 56 718.}	4202.43	1.3479	newly 5 weeks spec	43.	1110.03	{20., 1786.}	2499.6	1.03377	newly 5 weeks cumul	740.	29 290.3	{2311., 81 632.}	3858.15	1.33199	newly 50 weeks cumul	804.	32 050.1	{2562., 86 436.}	3886.33	1.35207
48	newly 3 weeks spec	186.	1544.96	{8., 2640.}	801.29	0.826792	newly 3 weeks cumul	969.	16 749.	{5690., 41 849.}	1633.34	0.999694	newly 5 weeks spec	49.	500.28	{0., 1281.}	1030.16	1.57814	newly 5 weeks cumul	1115.	18 108.4	{5737., 54 060.}	1528.14	0.962373	newly 50 weeks cumul	1182.	19 385.2	{5739., 57 924.}	1542.04	0.953336
49	newly 3 weeks spec	8.	3175.26	{217., 7211.}	39 590.8	2.11031	newly 3 weeks cumul	68.	11 973.7	{1327., 19 580.}	17 508.4	1.92225	newly 5 weeks spec	3.	1243.15	{57., 2933.}	41 350.3	2.11459	newly 5 weeks cumul	72.	15 312.7	{1635., 27 883.}	21 167.6	1.98904	newly 52 weeks cumul	76.	16 892.4	{1832., 30 686.}	22 126.9	2.01461
50	newly 3 weeks spec	733.	1301.22	{0., 3216.}	169.512	0.907277	newly 3 weeks cumul	2944.	11 153.4	{3902., 34 686.}	282.952	0.367124	newly 5 weeks spec	408.	267.35	{0., 410.}	117.934	1.33999	newly 5 weeks cumul	3889.	11 996.7	{3927., 36 306.}	213.885	0.27578	newly 43 weeks cumul	4985.	12 264.	{3927., 36 571.}	165.325	0.243911
